# A New Single-Step PCR Assay for the Detection of the Zoonotic Malaria Parasite *Plasmodium knowlesi*

**DOI:** 10.1371/journal.pone.0031848

**Published:** 2012-02-20

**Authors:** Naomi W. Lucchi, Mitra Poorak, Jenna Oberstaller, Jeremy DeBarry, Ganesh Srinivasamoorthy, Ira Goldman, Maniphet Xayavong, Alexandre J. da Silva, David S. Peterson, John W. Barnwell, Jessica Kissinger, Venkatachalam Udhayakumar

**Affiliations:** 1 Atlanta Research and Education Foundation, Decatur, Georgia, United States of America; 2 Malaria Branch, Division of Parasitic Diseases and Malaria, Center for Global Health, Centers for Disease Control and Prevention, Atlanta, Georgia, United States of America; 3 Department of Genetics, University of Georgia, Athens, Georgia, United States of America; 4 Center for Tropical and Emerging Global Diseases, University of Georgia, Athens, Georgia, United States of America; 5 Institute of Bioinformatics, University of Georgia, Athens, Georgia, United States of America; Université Pierre et Marie Curie, France

## Abstract

**Background:**

Recent studies in Southeast Asia have demonstrated substantial zoonotic transmission of *Plasmodium knowlesi* to humans. Microscopically, *P. knowlesi* exhibits several stage-dependent morphological similarities to *P. malariae* and *P. falciparum*. These similarities often lead to misdiagnosis of *P. knowlesi* as either *P. malariae* or *P. falciparum* and PCR-based molecular diagnostic tests are required to accurately detect *P. knowlesi* in humans. The most commonly used PCR test has been found to give false positive results, especially with a proportion of *P. vivax* isolates. To address the need for more sensitive and specific diagnostic tests for the accurate diagnosis of *P. knowlesi*, we report development of a new single-step PCR assay that uses novel genomic targets to accurately detect this infection.

**Methodology and Significant Findings:**

We have developed a bioinformatics approach to search the available malaria parasite genome database for the identification of suitable DNA sequences relevant for molecular diagnostic tests. Using this approach, we have identified multi-copy DNA sequences distributed in the *P. knowlesi* genome. We designed and tested several novel primers specific to new target sequences in a single-tube, non-nested PCR assay and identified one set of primers that accurately detects *P. knowlesi*. We show that this primer set has 100% specificity for the detection of *P. knowlesi* using three different strains (Nuri, H, and Hackeri), and one human case of malaria caused by *P. knowlesi*. This test did not show cross reactivity with any of the four human malaria parasite species including 11 different strains of *P. vivax* as well as 5 additional species of simian malaria parasites.

**Conclusions:**

The new PCR assay based on novel *P. knowlesi* genomic sequence targets was able to accurately detect *P. knowlesi*. Additional laboratory and field-based testing of this assay will be necessary to further validate its utility for clinical diagnosis of *P. knowlesi*.

## Introduction

Until recently, only four *Plasmodium* species, *P. falciparum*, *P. vivax*, *P. malariae* and *P. ovale*, were thought to contribute to human malaria infections. However, recent studies in Southeast Asia have shown zoonotic transmission of *P. knowlesi* to humans [1]–[15]. *P. knowlesi* is a parasite species that readily infects Old World monkeys, reviewed in [16]. The natural hosts of this simian malaria parasite are the long-tailed (*Macaca facsicularis*) and pig-tailed (*M. nemestrina*) macaque monkeys and langurs (*Presbytis* sp.) [17]–[19] that are distributed throughout much of Southeast Asia. The transmission of *P. knowlesi* is closely related to its vector species in the *Anopheles leucophyrus* group, which are forest-dwelling mosquitoes found in forest canopies or on forest fringes [8]–[10]. Indeed, many of the reported human *P. knowlesi* cases were found either near forests or as imported cases from individuals known to have visited the forests [20]–[22]. To date, no human-to-human transmission has been documented and chloroquine is effective in treating these infections [3]. *P. knowlesi* has a 24-hour asexual life cycle [23], the shortest observed, thus far, for human-infecting parasites. This short cycle can lead to rapid increases in parasitemia and can lead to severe disease including fatalities as reported in recent studies [1], [2]. Given these observations, human infections with *P. knowlesi* require immediate and appropriate treatment, which in turn depends upon a prompt and accurate diagnosis.

**Figure 1:**
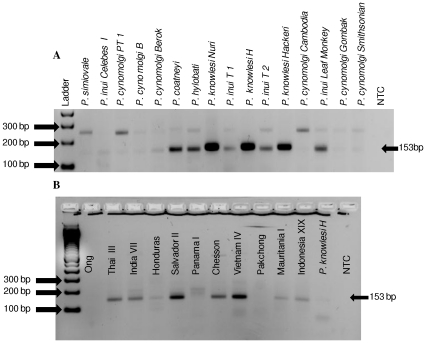
18S ribosomal RNA gene based *P. knowlesi* primer cross-reacts with *P. vivax* and other simian-infecting malaria species. Published 18S ribosomal RNA gene based *P. knowlesi* primers [3] were used to test 5 different simian-infecting malaria parasite species (*P. simiovale*, *P. inui*, *P. cynomolgi*, *P. hylobati and P. coatneyi*) including 3 different *P. knowlesi* isolates (A) and 11 *P. vivax* strains (B). A no template control (NTC) was included. Cross reactivity was observed with some of the simian malaria parasites and some *P. vivax* strains.

Microscopically, *P. knowlesi* exhibits stage-dependent morphological similarities to *P. malariae* and *P. falciparum* [3], [24]. These similarities have contributed to misdiagnosis of *P. knowlesi* as *P. malariae* [1], [3] or *P. falciparum*. For example, a study in the Kapit Division of Malaysian Borneo, found that 58% of previously diagnosed *P. malariae* cases were actually *P. knowlesi* infections [3]. In this study by Singh et al., [3] a nested PCR-based diagnostic test for the detection of *P. knowlesi* 18S ribosomal RNA genes was developed and has been used in numerous subsequent studies [1], [7]–[9], [25]–[27]. However, this test was recently noted to cross-react with *P. vivax* leading to potential false positive results for a small proportion of human clinical *P. vivax* samples [26]. This observation was confirmed by results from our laboratory, in which cross reactivity with *P. vivax* and other simian *Plasmodium* species (*P. cynomolgi*, *P. inui*, *P. coatneyi*, and *P. hylobati*) was observed (Figure 1). These findings have raised some concern about the actual extent of the reported *P. knowlesi* cases [28], [29], although *P. knowlesi* DNA from some of the diagnosed cases was sequenced in order to confirm the presence of this parasite [7]–[9]. Therefore, development of an improved molecular diagnostic test is critical not only for the proper diagnosis of human infections, but also for estimating the true burden of *P. knowlesi* infection in human populations.

Imwong *et al*. recently reported a nested PCR assay with 100% specificity for detecting *P. knowlesi* [26]. In addition, a loop mediated isothermal amplification (LAMP) method designed to detect the beta tubulin gene of *P. knowlesi* [30] and two real-time PCR assays [31], [32] have been reported to be highly specific for the detection of *P. knowlesi.* We recently reported on the use of a bioinformatics approach to mine available genome data and identify suitable DNA sequences that are highly specific to a given species of malaria parasite [33]. Using this approach, we have identified highly-specific, multi-copy sequences from the *P. knowlesi* genome and designed novel primers that can be used in a single-tube, non-nested PCR diagnostic test. We have identified one set of primers that has high specificity (100%) for the detection of *P. knowlesi* at a low level of parasitemia (1 parasite per uL).

## Methods

### *Plasmodium* parasites and clinical samples

Different *Plasmodium* species available in our laboratories were utilized to test the specificity of the novel *P. knowlesi* primers: *P. falciparum* (3D7 clone), *P. vivax* (South Vietnam IV), *P. malariae* (Uganda I), *P. ovale* (Nigeria I), and 11 other *P. vivax* strains (Ong, Thai III, India VII, Honduras I, Salvador II, Panama I, Chesson, Vietnam IV, Pakchong, Mauritania I and Indonesia XIX). Three *P. knowlesi* isolates (Nuri, H, and Hackeri) and 5 simian malaria parasites (*P. simiovale*, *P. inui*, *P. cynomolgi*, *P. hylobati* and *P. coatneyi*) available in the CDC laboratory collection were included. In addition, DNA from 52 clinical samples, previously diagnosed using a nested PCR method [34] (14 *P. falciparum*, 9 *P. vivax*, 1 *P. malariae*, 12 *P. ovale*, *2 P. falciparum/P. malariae*, *1 P. vivax/P. ovale*, *2 P. falciparum/P. ovale* mixed infections, 1 *P. knowlesi* and 10 malaria negative samples), were tested in a blinded manner. The *P. knowlesi* sample was acquired from a traveler who returned infected after a trip to the Philippines in 2008, representing the first recognized case of imported simian malaria in several decades in the United States [35]. These clinical samples were obtained from the CDC molecular diagnostic parasitology reference laboratory (Dr. A. da Silva).

### DNA extraction

The QIAamp DNA Mini Kit (QIAGEN, Valencia, CA-(Qiagen method) was used to isolate DNA from the different *Plasmodium* parasites following the manufacturer's protocol. The DNA was aliquoted and stored at −20°C until used in the experiments.

### Novel *P. knowlesi* target validation

Assembled genome sequence data for *P. knowlesi* was obtained from PlasmoDB (http://plasmodb.org/plasmo/; release 5.5). The sequence candidates were selected as previously described [33]. Briefly, genome sequence data were mined for repetitive content. The identified repetitive sequences were screened for a number of properties that would negate their utility as PCR targets, such as tandem repeats and human or artificial (vector) sequence similarity. Repeats passing these screens were evaluated for species-specificity. The copy number of candidate targets satisfying a length requirement of 300 bp was determined, and targets with greater than 5 copies/genome were further considered as potential diagnostic targets. Primers were designed manually to the candidate targets and screened for GC-content, melting temperature, secondary structure, and primer-dimer forming potential. Primer pairs were optimized by means of gradient PCR using *P. knowlesi* DNA (strain H) to determine the optimum annealing temperature, primer concentration (concentrations from 0.25 µM to 1.0 µM were tested) and MgCl_2_ concentrations (2.0mM–4.0mM were tested). Primers were then tested for *P. knowlesi* specificity and sensitivity.

### Specificity assay

Primers that passed the initial validation tests were further tested for specificity using 11 *P. vivax* strains and different simian *Plasmodium* species and strains. DNAs from 52 clinical samples were tested blindly.

### PCR assays

All PCR tests were completed on a BioRad iCycler (BioRad, Hercules, CA). Nested PCR for *P. knowlesi* was performed with primers and cycling conditions as described before [3]. The confirmatory nested PCR used to test the 52 clinical samples was as previously described [34]. The PCR amplified material was analyzed using gel electrophoresis (2% agarose gel) to visualize the bands of appropriate size. Amplification of *P. knowlesi* using the novel primers was performed in a 25 µl reaction containing 1× Taq Buffer (containing 10mM Tris-HCl, 50mM KCl, 1.5mM MgCl_2_) , 200 µM each dNTP, 1.25 units of Taq DNA Polymerase (all from New England Biolabs, Ipswich MA, USA), 250nM each oligonucleotide primer, and 1 µl of DNA template. The sequences of the final oligonucleotide primer set (Pkr140-5) selected for *P. knowlesi* detection are shown in Table 1. Reactions were performed under the following cycling parameters: initial denaturation at 95°C for 2 minutes, and then 35 cycles of 95°C for 30 seconds, 57°C for 30 seconds, and 72°C for 45 seconds, followed by final extension at 72°C for 5 minutes. Ten µL of PCR products were visualized by gel electrophoresis on a 2% agarose gel.

**Table 1:** Sequence of the novel Pkr140-5 primer set.

Primer	Sequence
**Forward**	5′- CAGAGATCCGTTCTCATGATTTCCATGG -3′
**Reverse**	5′- CTRAACACCTCATGTCGTGGTAG-3′

The primers were designed to target Pkr140, which is present in 7 copies distributed across 6 different chromosomes in the available genome sequence.

### Limits of detection of the PCR amplification using the new primers

The analytical sensitivity of the assay was determined using a well-quantified *P. knowlesi* H strain sample obtained from an infected monkey. The WHO recommended protocol for the preparation of standards for use in the quality control of rapid diagnostic tests (http://www.wpro.who.int/sites/rdt/using_rdts/qa/lot_testing.htm) was used to prepare the parasite standard for this study. The *P. knowlesi* parasites were at either the ring or early trophozoite stages of development when the sample was utilized. The percent parasitemia of the infected monkey was determined by three expert microscopists by counting the number of infected erythrocytes in 10,000 erythrocytes. The total number of erythrocytes per microliter was determined through use of a coulter counter and the number of parasites/µL was then determined from the total number of RBCs/µL. The resulting parasitemia was determined to be 225,000 parasites/µL. This standard sample was then diluted from the initial parasitemia to 100,000 parasites/µL using uninfected blood and then serial diluted ten-fold to 1p/µL using a 250 µL volume. DNA was extracted from each dilution point using 200 µL of sample. These diluted samples were used to test the limits of detection of the previously described primers [3] and the novel Pkr140-5 primer set described here.

## Results

### Primer Design

Four genomic sequence targets passed the *in silico* tests and were selected for validation. A total of 14 primers were designed to these targets and empirically tested in conventional PCR amplification assays using *P. knowlesi*-H DNA sample. Of the 14 primers designed, three sets (Pkr140-3, Pkr140-4 and Pkr140-5), all of which recognize the Pkr140 repeat sequence, were selected for further evaluation as they correctly amplified *P. knowlesi* as evidenced by clean, intense, single bands of the expected size. The Pkr140 sequence exists in 7 copies in the available *P. knowlesi* genome sequence.

### Tests for assay specificity

**Figure 2:**
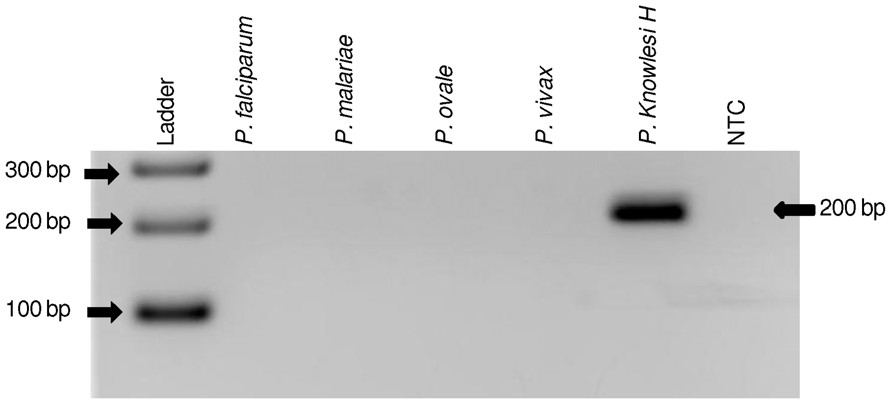
Primer Pkr140-5 tested with the 4 human-infecting malaria species. To test the specificity of the novel primers, DNA from the four additional human-infecting *Plasmodium* parasites were tested. *P. knowlesi* (H strain) was used as a positive control (expected size = 200bp). A no template control (NTC) was also included.

**Figure 3:**
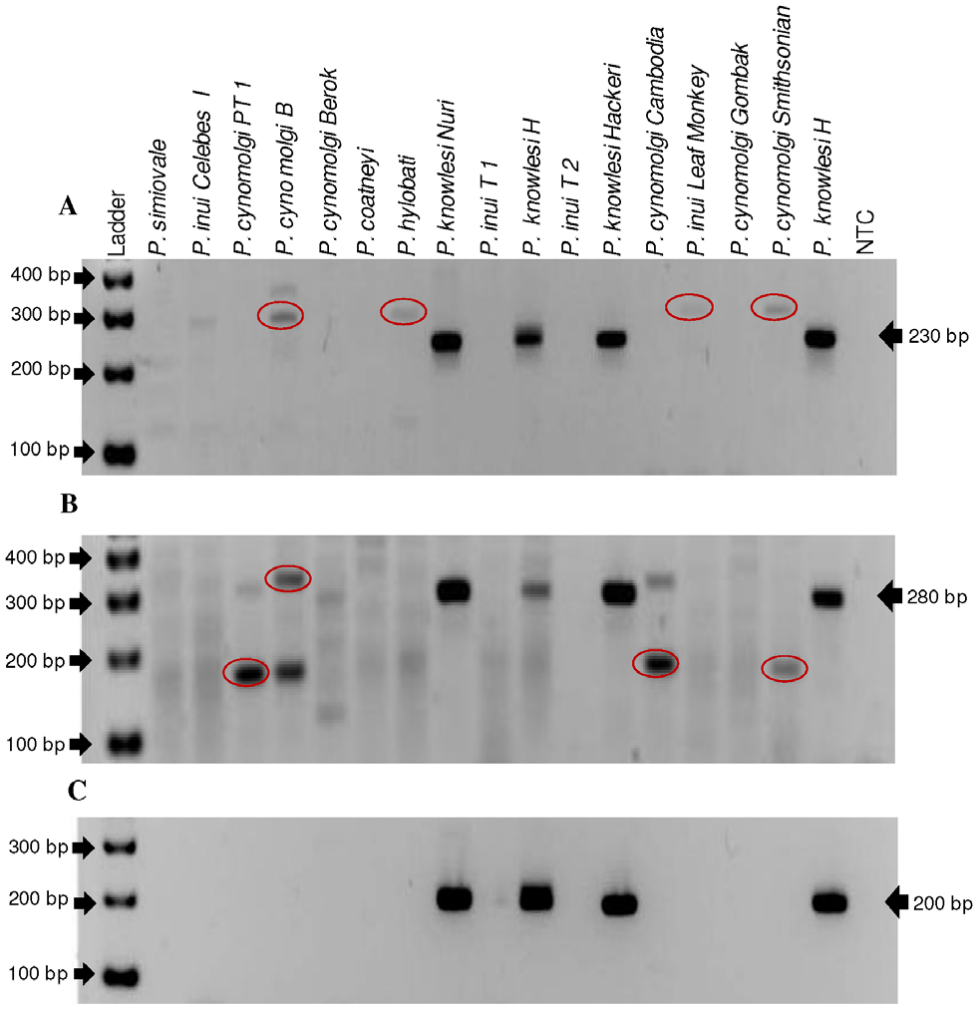
Specificity of the *P. knowlesi* primers tested using simian-infecting malaria species. To test the specificity of the *P. knowlesi* primers, 5 different simian-infecting malaria parasite species (*P. simiovale*, *P. inui*, *P. cynomolgi*, *P. hylobati and P. coatneyi*) including 3 different *P. knowlesi* isolates were tested. The no template control (NTC) was included as a negative control. A; primer set Pkr140-3 (expected size = 230bp), B; primer set Pkr140-4 (expected size = 280bp) and C; primer set Pkr140-5 (expected size = 200bp). Circles indicate non-specific amplification.

**Figure 4:**
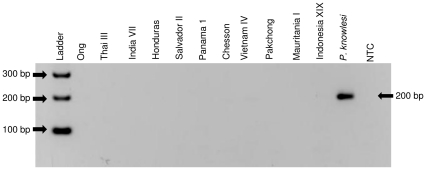
Primer set Pkr140-5 does not cross react with *P. vivax*. Multiple *P. vivax* strains were tested using primer set Pkr140-5 (expected size 200bp) in order to test the primers' specificity. NTC = No template control.

The three Pkr140 primer sets were tested for species-specificity initially using DNA from *P. falciparum*, *P. vivax*, *P. ovale* and *P. malariae.* No cross-reactivity was observed with these species (Figure 2 and data not shown) as there was no amplification of these DNA. Second, the primers were tested for specificity against 5 different simian malaria parasites (*P. simiovale*, *P. inui*, *P. cynomolgi*, *P. hylobati* and *P. coatneyi*) and strains thereof. Primer set Pkr140-3 produced non-specific bands with *P. inui*, *P. cynomolgi*, and *P. hylobati* (Figure 3A) and primer set Pkr140-4 with *P. cynomolgi* (Figure 3B). These two primer sets were not evaluated further. Primer set Pkr140-5 (Table 1) detected only the three *P. knowlesi* isolates (H, Nuri, and Hackeri) used in this study (Figure 3C) and did not amplify DNA from any of the eleven *P. vivax* isolates tested (Figure 4).

### Further test for specificity using clinical samples

Forty two DNA specimens extracted from clinical samples previously confirmed by PCR as positive for malaria (14 *P. falciparum*, 9 *P. vivax*, 1 *P. malariae*, 12 *P. ovale*, *2 P. falciparum/P. malariae*, *1 P. vivax/P. ovale*, *2 P. falciparum/P. ovale* mixed infections, 1 *P. knowlesi*) and 10 malaria negative clinical samples were further used to test the specificity of primer set Pkr140-5 in a blinded manner. This primer set correctly identified the single sample with known *P. knowlesi* infection [35] and did not show any cross-reactivity with any of the other samples.

### Limits of detection of primer set Pkr140-5

**Figure 5:**
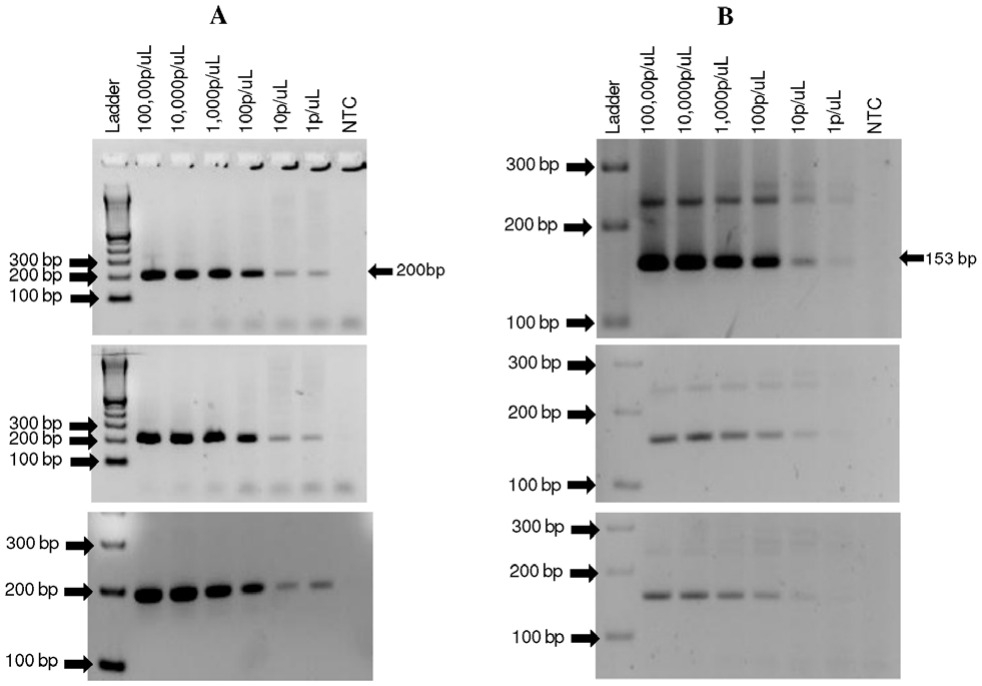
Limits of detection of primer set Pkr140-5. The analytical sensitivity of primer set Pk140-5 (A) and the primers from a published study [3] (B) were determined using a well-quantitated *P. knowlesi* DNA standard. The blood sample was serially diluted ten-fold with a starting parasitemia of 100,000p/µl to 1p/µl. The expected base pair sizes for the two primers are included. Three different experiments are shown.

Using known quantities of *P. knowlesi*-H DNA, both the previously published *P. knowlesi* primers and the novel Pkr140-5 primer set were able to detect up to 1 parasite of *P. knowlesi*/µL of blood with the novel primer set showing better resolution than the previously published primer set (Figure 5).

### Characteristics of Pkr140

**Figure 6:**
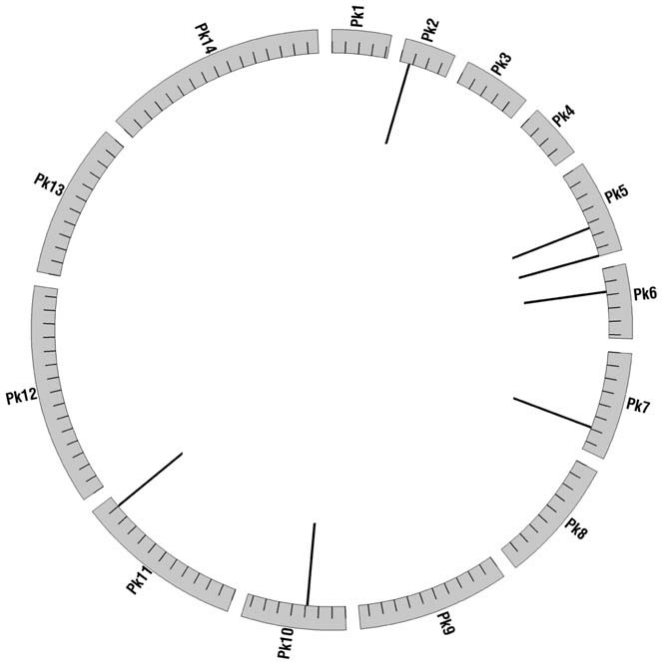
Spatial distribution of Pkr140 sequence targets across the 14 *P. knowlesi* genome. The circle represents chromosomes. Each chromosome is labeled with the 2-letter genus and species abbreviation for *P. knowlesi* and the chromosome number. Tick marks indicate 1 mb of sequence. Lines inside the circle indicate the location of Pkr140 copies and are not to scale. Circos 0.51 (http://mkweb.bcgsc.ca/circos/) was used to generate this map [44].

The Pkr140 sequence repeats are present in 7 copies distributed across 6 chromosomes (Figure 6). Six of the copies have an average size of 424 bps. The seventh copy (closest to the end of chromosome 5) is truncated (only 42 bps) and is not amplified by primer set Pkr140-5. We previously identified repetitive sequence targets in *P. falciparum and P. vivax* that were distributed to subtelomeric regions or to contigs thought to belong to subtelomeric regions [33]. In contrast, the Pkr140 sequences are found both near chromosome ends and interior regions. The Pkr140 sequences do not appear to be protein-encoding and they have not been annotated as serving any particular function. Moreover, searches of PlasmoDB (http://plasmodb.org) did not reveal any possible function for these sequences. Interestingly, 5 of the 7 Pkr140 repeat sequences (including the truncated copy) are located near genes that encode the *SICAvar* antigen, a member of one of the main variant gene families in *P. knowlesi* [36], [37]

## Discussion

In this study, we report a new PCR assay based on novel genomic target sequences for *P. knowlesi* detection. We have previously reported on the use of a bioinformatics method to mine parasite genome sequences in search of species-specific and multi-copy sequences that can be used to design diagnostic PCR primers for malaria detection [33]. Using this genome-mining approach, 14 primer sets were designed and tested for their utility for *P. knowlesi* detection in a non-nested PCR assay. Three sets of primers were found to amplify *P. knowlesi* consistently. However, two of these sets produced non-specific bands with some simian malaria parasites and were not tested further as our goal was to identify primers that specifically amplify *P. knowlesi*. We identified primer set Pkr140-5 as specific for the detection of *P. knowlesi* as it did not detect any other human malaria parasites nor any of the five simian malaria species tested, including the closely related species *P. inui and P. cynomolgi.*

Previously identified diagnostic targets in *P. falciparum* and *P. vivax* [33] were distributed at chromosome ends or unassembled contigs belonging to chromosome ends. Subtelomeric regions in these species have been shown (to varying degrees) to be enriched for species-specific and multi-copy genes [38] and genes involved in antigen variation [39], [40]. The *P. knowlesi* genome organization differs from *P. falciparum* and *P. vivax* with genes involved in antigenic variation distributed across chromosomes and not concentrated at their ends. Given this difference in genome organization, and the proximity of the identified Pkr140 targets to *SICAvar* genes, it is perhaps not surprising that the targets are also distributed across both chromosome ends and interiors. Based on the results from three *Plasmodium* species, regions near multi-gene families are potentially rich areas for the mining of diagnostic targets.

Our data, reported here, further confirms a previous report of cross reactivity between 18S ribosomal RNA gene primers [3] and *P. vivax* parasites. In addition, our results demonstrate that the 18S ribosomal RNA gene primers also cross-react with at least four simian malaria parasites (*P. inui*, *P. hylobati*, *P. cynomolgi*, and *P. coatneyi*). The difficulty of *P. knowlesi* diagnosis with the 18S ribosomal RNA gene-based PCR assay was also recently highlighted in a study in which 2 samples determined to be positive for *P. knowlesi* could not be confirmed by DNA sequencing analysis [41]. The primer set described here showed 100% specificity and no cross reactivity observed with any of the non- *P. knowlesi* samples tested. In addition, this primer set showed a limit of detection of 1 parasite/µL which was shown to be comparable to the limits of detection of the previously described nested PCR test [3]. This is promising as the primer set can be used for the detection of low parasite levels without the need to perform a nested PCR. A limitation of the current study is the fact that only one clinical *P. knowlesi* sample was available for use to test the novel primer sets; however, three *P. knowlesi* strains obtained from monkeys were included to validate the specificity. Given the fact that the occurrence of human *P. knowlesi* is a pretty novel phenomenon that is rather confined mainly in Southeast Asia, it was not immediately possible to evaluate a large number of *P. knowlesi* samples. Therefore, further validation of these primers in regions known to have *P. knowlesi* transmission will be required to test their utility for *P. knowlesi* diagnosis. However, the lack of a large sample size does not negate the fact that these primers are indeed specific and sensitive to detect *P. knowlesi*.

Molecular tools for *P. knowlesi* detection have been reported including nested PCR assays, two real-time PCR assays and a loop mediated isothermal amplification (LAMP) assay [30]–[32], [42]. The PCR test described here does not require nested amplification, simplifying the performance of the reaction and saving on costs. The LAMP assay holds potential for use in regions with limited or fewer resources, as it does not necessitate the use of expensive thermal cyclers. The real-time PCR assays' use is limited to settings with real-time PCR capabilities such as reference laboratories. It remains to be determined if these different assays vary in their sensitivity and specificity to diagnose *P. knowlesi* infection in field/clinical settings.

Human *P. knowlesi* infections have been mostly reported in Southeast Asia [1]–[15]. Recently, several imported cases in other parts of the world have also been reported [20]–[22], [43] including the United States [35]. The novel non-nested PCR assay described in this study is a suitable alternative for the accurate diagnosis of *P. knowlesi* by PCR in most laboratories. However, additional laboratory and field-based testing of this assay will be necessary to validate its utility for clinical diagnosis of *P. knowlesi.*
